# Emergency and prophylactic uterine artery embolization in gynecology and obstetrics - a retrospective analysis

**DOI:** 10.2478/raon-2024-0037

**Published:** 2024-09-15

**Authors:** Polona Vihtelic, Eva Skuk, Natasa Kenda Suster, Marina Jakimovska Stefanovska, Peter Popovic

**Affiliations:** Clinical Institute of Radiology, University Medical Centre Ljubljana, Ljubljana, Slovenia; Faculty of Medicine, University of Ljubljana, Ljubljana, Slovenia; Division of Obstetrics and Gynecology, University Medical Centre Ljubljana, Ljubljana, Slovenia

**Keywords:** postpartum hemorrhage, retained products of conception, endovascular treatment, uterine artery embolization, hysterectomy, fertility

## Abstract

**Background:**

This study aimed to evaluate the safety and efficacy of emergency and prophylactic uterine artery embolization (UAE) in our clinical practice, including technical success, clinical success, and associated complications.

**Patients and methods:**

In this retrospective study, we analyzed 64 women who underwent emergency (*n* =18) and prophylactic (*n* = 46) UAE. Indications for emergency UAE included postpartum hemorrhage or severe hemorrhage during pregnancy termination, while prophylactic UAE was performed prior to surgical removal of retained products of conception (RPOC), delivery with abnormal placental implantation, or pregnancy termination (cervical pregnancy or fetal anomalies accompanied by abnormal placental implantation). Technical success of UAE was defined as complete exclusion of the vascular lesion and contrast stasis on the final angiogram, while clinical success was defined as cessation of bleeding after UAE Termination without a hysterectomy.

**Results:**

The overall clinical success of UAE in our study was 97% (62/64). All embolization procedures were technically and clinically successful in the prophylactic group without life-threatening hemorrhages or hysterectomies (100% success rate, 46/46). However, while 100% technical success was similarly attained in the emergency group, bleeding was successfully controlled in 89% of cases (16/18). In two patients with significant blood loss (over 2000 mL), embolization failed to achieve hemostasis, resulting in persistent bleeding and subsequent hysterectomy.

**Conclusions:**

UAE is a safe and effective procedure for managing primary postpartum hemorrhage or severe hemorrhage during pregnancy termination and for decreasing the risk of severe hemorrhage during surgical removal of RPOC, delivery with abnormal placental implantation, or pregnancy

## Introduction

Complications during pregnancy include severe life-threatening hemorrhage that can occur during vaginal or cesarean delivery, pregnancy termination, or surgical removal of retained products of conception (RPOC).

Primary postpartum hemorrhage (PPH) is a leading cause of obstetric morbidity and mortality globally, accounting for more than 100,000 maternal deaths annually. Pathologic postpartum hemorrhage, which occurs in up to 10% of deliveries, is characterized by excessive blood loss during childbirth, defined as more than 500 mL for vaginal deliveries and more than 1,000 mL for cesarean births. Postpartum hemorrhage is further classified based on the time of its occurrence. Primary postpartum hemorrhage refers to excessive bleeding within 24 hours after delivery, whereas secondary postpartum hemorrhage describes bleeding that occurs anytime within six weeks after birth.^1,2,3,4^ The most common causes of primary PPH are summarized in the 4 T mnemonics and include uterine a**t**ony, **t**rauma (birth canal lacerations), **t**issue (retained/abnormal placenta), and **t**hrombin (coagulopathies). Uterine atony accounts for approximately 70% of cases of PPH and can lead to hemorrhagic shock, which may progress to endothelial damage and disseminated intravascular coagulation (DIC).^1,2,3,4,5^ Placental causes, including abruption or placental implantation abnormalities (*placenta previa, accreta, percreta*, *increta*), are less frequent conditions but can also lead to severe postpartum hemorrhage.^2,3^

Retained products of conception (RPOC) refer to persistent placental and/or fetal tissue that remains in the uterine cavity after a vaginal or cesarean delivery, miscarriage, or pregnancy termination.^6,7,8,9^ The estimated prevalence of RPOC is approximately 1% in term pregnancies and is more frequent after medical termination of pregnancy or miscarriage.^8^ Recent literature reports a newly identified form of RPOC with highly vascularized characteristics on Doppler ultrasound observations. According to the literature, the occurrence rate of this particular entity is estimated to be around 18%.^6^ RPOC is associated with long-term complications, such as intrauterine adhesions, menstrual abnormalities, infertility, recurrent pregnancy loss, and placental complications.^3,6,7,8^

Hemorrhage during pregnancy termination occurs in less than 1% of abortions. Bleeding may result from uterine atony, placental abnormalities spectrum, lacerations, and coagulopathies.^3,9^

Cervical pregnancy is a rare form of ectopic pregnancy, where pregnancy implants in the endocervical canal, and it accounts for less than 1% of all ectopic pregnancies. The incidence is estimated to be 1 in 9,000 pregnancies. The condition requires pregnancy termination, and the main potential complication is a high risk of severe hemorrhage.^10,11^

**Figure 1.**
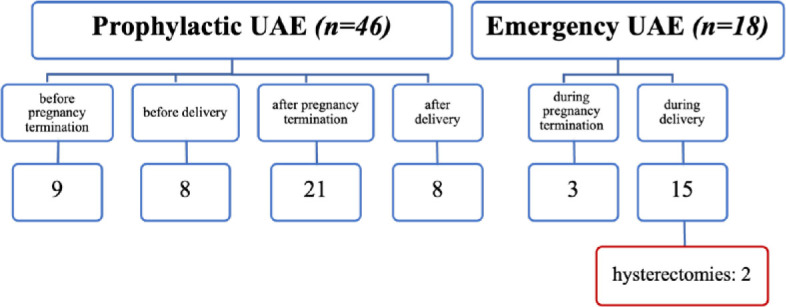
Patient distribution for prophylactic and emergency uterine artery embolization (UAE).

Selective transcatheter uterine artery embolization was first reported in 1979 as a second-line therapy for severe persistent postpartum hemorrhage.^4^ Subsequent publications have further established the efficacy and safety of endovascular methods in controlling uterine bleeding and achieving hemostasis.^1,2,3,4,5^ In 2017, the American College of Obstetricians and Gynecologists incorporated transcatheter arterial embolization (TAE) into their recommendations for managing postpartum hemorrhage, emphasizing the importance of preserving the uterus and potentially future fertility. Additionally, the International Federation of Gynecology and Obstetrics recognized the safety and efficacy of TAE in their 2022 PPH management guidelines, stating that this technique is a viable option for patients prioritizing fertility preservation.^4^

Embolic agents include absorbable gelatin sponges, microspheres, liquid agents, and cyanoacrylate glue. The preferred embolic material in our practice is the gelatin sponge, as this is the most frequently studied embolic agent in the treatment of postpartum hemorrhage, which temporarily (3 – 6 weeks) occludes the target vessel. Subsequent recanalization of the arteries has a theoretical advantage for patients desiring future fertility.^1,5^

### Imaging findings

PPH and pregnancy termination-related hemorrhage are conditions that require immediate intervention; therefore, imaging is often limited to digital subtraction angiography during an endovascular procedure. Computer tomography angiography (CTA) is routinely not performed in the preprocedural assessment of postpartum hemorrhage due to time delay in the setting of active bleeding and radiation exposure to radiosensitive tissue in a typically young patient. However, CTA can be used when the underlying cause or location of hemorrhage is uncertain or if there is a recurrence of bleeding after an initially successful embolization.^12^

**Figure 2.**
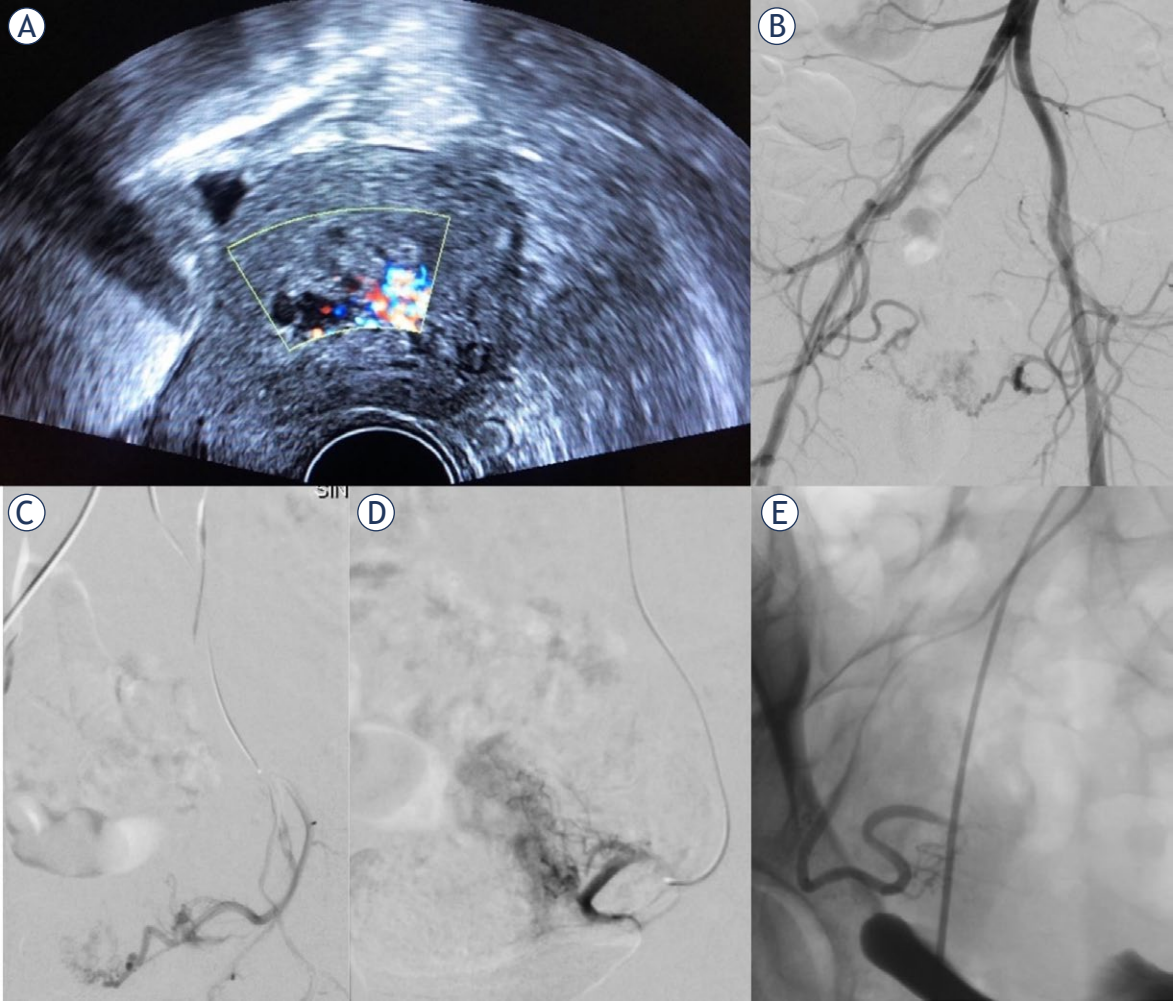
(A) Transvaginal ultrasonography in a 34-year-old female with vaginal bleeding after spontaneous termination of pregnancy at 12 weeks of gestation. Color Doppler ultrasound showed a 45 x 30 mm mass in the uterus with increased vascularity suggestive of retained products of conception. **(B)** Pelvic arteriogram demonstrating numerous spiral arteries in the uterus fed by both right and left uterine arteries, confirming the diagnosis of RPOC. **(C)** Left uterine arteriogram in the same patient before prophylactic embolization with absorbable gelatin sponge particles showing numerous spiral arteries. Postembolization left **(D)** and right **(E)** uterine arteriogram demonstrating successful embolization. Subsequently, surgical resection of retained products of conception was successfully performed.

Doppler ultrasound and magnetic resonance imaging can identify placental irregularities, cervical pregnancy, and vascularization in the case of RPOC. These two imaging techniques are often required in planning delivery and the proper treatment protocol for pregnancy termination or RPOC.^11,12,13^ In the case of RPOC, ultrasound helps us to assess the degree of vascularization. The marked vascularity feature is established in the presence of an exuberant color Doppler signal (Figure 2A).^6,7,8^

**Figure 3.**
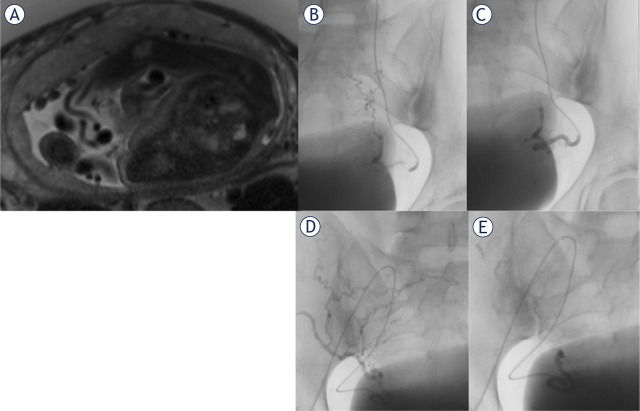
(A) Abdominal magnetic resonance imaging in a 35-year-old woman with singleton pregnancy at the 33rd week of gestation with suspected placenta accrete. The placenta covers the whole anterior part of the uterine wall. On T2-weighted MR images, there is marked thinning of the myometrium at the placental-myometrial interface, with MRI signs of placenta accrete and placenta increta. On the left cranial part of the junction, there is suspected placental invasion through the entire uterine wall, suspicious for placenta percreta. **(B-E)** Selective uterine angiography and embolization following delivery in the same 35-year-old woman with placenta accrete. Left **(B)** and right **(D)** uterine angiogram confirming the diagnosis of RPOC with numerous voluminous spiral arteries before prophylactic embolization. Left **(C)** and right **(E)** post-embolization angiogram after successful selective uterine artery embolization with absorbable gelatin sponge particles to reduce vascularity prior to surgical removal. After embolization, the placental tissue was successfully resected, and hysterectomy was prevented.

Contrast-enhanced CT has a limited role in the evaluation of hypervascular RPOC due to inaccuracy in soft tissue assessment. RPOC on CT shows an intensely enhanced heterogenous mass in the uterine cavity during the arterial phase or extravasation of intravenous contrast in case of large arterial hemorrhage in the postpartum period.^14-15^ Pelvic MRI provides more detailed information if the diagnosis of hypervascularity in RPOC is unclear and there is no urgency.^7,14^ Key MRI findings include intracavitary uterine soft-tissue mass with variable T1 and T2 signal intensities, variable amounts of enhancing tissue, and variable degrees of myometrial thinning and obliteration of the junctional zone (Figure 3A).^14,16^

Digital subtraction angiography (DSA) is the gold standard for evaluating blood vessels and confirming intrauterine vascular lesions.^6^ Angiographic findings include enlarged and tortuous uterine arteries with well-defined focal masses made up of vascular tangles, a focal blush of contrast, early venous return, extravasation, and pseudoaneurysm (Figures 2B, D; 3B, D).^6,8^ However, the presence of active extravasation on DSA is relatively rare in cases of postpartum hemorrhage, particularly in cases of uterine atony. The required rate of bleeding for angiographic detection is typically around 1–2 mL/min, which may be too low to detect in cases of uterine atony.^17^

### Treatment options

Severe acute uterine cavity bleeding during vaginal/cesarean delivery, pregnancy termination, and removal of RPOC is initially managed by complete emptying of the contents of the uterine cavity, uterine massage, uterotonics, fluid resuscitation, and tranexamic acid administration. In some patients, further mechanical (intrauterine balloon tamponade) or sometimes surgical (internal iliac artery ligation) interventions are required.^18^ Hysterectomy has traditionally been considered the last and definitive treatment option.^4,12,13^ To avoid the sterility and significant morbidity associated with emergent hysterectomy, uterine artery embolization has been emphasized as a minimally invasive therapy for rapidly controlling hemorrhage that is refractory to standard gynecological treatment.^5^

In some cases of uterine hemorrhage, immediate hemodynamic instability is not a predominant concern, but according to imaging findings (ultrasound and MRI), a high risk for severe hemorrhage is expected with further gynecological intervention. Such cases may include vaginal/cesarean delivery with abnormal placentation, pregnancy termination with abnormal placentation, termination of cervical pregnancy, and surgical removal of highly vascularized RPOC (Figure 2, 3).^3,6,7,12,13^ In these cases, prophylactic uterine artery embolization (UAE) is performed prior to surgical removal of RPOC, delivery, or pregnancy termination to reduce the risk of hemorrhage, offering a safe alternative to hysterectomy and, therefore, preserving fertility.^6,7,8^

## Patients and methods

### Study population

This retrospective single-center study was conducted at the University Medical Centre Ljubljana and was approved by the National Medical Ethics Committee of the Republic of Slovenia on December 8, 2021 (ref. no. 0120-285/2021/11). The study analyzed clinical data over a ten-year period (from 2012 to 2022). The data were obtained from the hospital information system of the University Medical Centre Ljubljana, the Perinatal Information system of the Republic of Slovenia, and from the completed patient questionnaires.

A total of 64 patients were included in the analysis and divided into two main categories – women who underwent prophylactic (46 patients) or emergency (18 patients) UAE. Patients in the prophylactic group were treated with UAE with the goal of decreasing the risk of massive bleeding during surgical removal of RPOC, pregnancy termination, or delivery. Patients in the emergency group were treated with UAE to control acute uterine cavity hemorrhage during pregnancy termination or delivery that was refractory to standard gynecological management. In both indications, the ultimate goal was to reduce blood loss and avoid an emergency hysterectomy.

The following data were collected from the medical records: age of patients, gynecological history, gestational week at pregnancy termination or delivery, indication for pregnancy termination, placental abnormalities, cause of PPH, type of UAE (prophylactic/emergency), estimated total blood loss during pregnancy termination, delivery or gynecological procedure and the necessity for hysterectomy.

The clinical diagnoses were made by a complete gynecological examination, followed by a sonographic assessment with a color Doppler ultrasound. Pelvic magnetic resonance imaging (MRI) was performed in indecisive cases and when there was no urgency. All patients underwent a diagnostic uterine digital subtraction angiography to confirm the diagnosis of intrauterine vascular lesion or hemorrhage before embolization. The decision on endovascular treatment was made by a multidisciplinary team, including gynecologists/obstetricians and interventional radiologists.

### UAE procedure

Pelvic angiography was performed via a transfemoral approach under local anesthesia, followed by a selective bilateral or unilateral uterine artery angiography (Figure 2, 3). In all cases, a 5-French sheath was inserted into the right common femoral artery, followed by catheterization and angiography of the internal iliac artery to identify the uterine arteries on both sides as well as potential sites of bleeding. Next, selective catheterization of uterine arteries with a microcatheter (Progreat, Terumo) was performed to decrease the risk of inducing vasospasm. Arterial feeder pedicles were embolized by absorbable gelatin sponge particles (Marbagelan), microspheres, and balloon occlusion at the level of the internal iliac artery. The embolization was completed after contrast stasis as determined by fluoroscopy and confirmed by bilateral uterine artery angiography.

### Study endpoints and definitions

This study aimed to evaluate the safety and efficacy of UAE in a cohort of 64 patients who underwent the procedure for the management of obstetric or gynecologic hemorrhage. The study focused on analyzing technical success, clinical success, and complications.

Pregnancy termination (also called abortion) was defined as the cessation of pregnancy before the 22^nd^ gestational week or when the fetus weighs less than 500 g. Conversely, the termination of pregnancy beyond the 22^nd^ gestational week was categorized as delivery.

Technical success was defined as the embolization of bilateral or unilateral uterine arteries with complete exclusion of the vascular lesion and contrast stasis on the final angiogram. Clinical success was defined as cessation of bleeding after UAE without a hysterectomy.

Postembolization complications were documented according to the criteria of the Society of Interventional Radiology.^19^ To confirm the efficacy of the interventional procedure and uterine surgical treatment, patients were carefully monitored on outpatient follow-up visits with referring obstetricians or gynecologists until confirmation of the absence of any re-bleeding or uterine irregularities.

## Results

The age range of the women included in the study was 22 – 45 years (mean 34 ± 6). Among them, 46 patients (72%) were treated with prophylactic UAE and 18 (28%) with emergency UAE.

In the prophylactic UAE group (Table 1), the patients were further subdivided into four subgroups based on both timing and cause of pregnancy termination and cause of RPOC.

**Table 1.** Clinical overview of the prophylactic uterine artery embolization (UAE) group

**PROPHYLACTIC UAE**	**Number of cases**	**Intervention**	**Blood loss during gynecological procedure**	**Hysterectomy**
**BEFORE PREGNANCY TERMINATION**				
Fetal anomalies accompanied by placental abnormalities	5	**Embolization** → pregnancy termination	300 –400 mL(median 300 mL)	**0**
Cervical pregnancy	4	**Embolization** → pregnancy termination	100 –400 mL (median 250 mL)	**0**
**BEFORE DELIVERY**				
Placental abnormalities with or without fetal anomalies	8	**Embolization** → vaginal/cesarean delivery	200 –1,800 mL (median 400 mL)	**0**
**AFTER PREGNANCY TERMINATION**				
Retained products of conception (RPOC)	21	**Embolization** → surgical removal of RPOC	100 – 400 mL	**0**
**AFTER DELIVERY**				
Retained products of conception RPOC	8	**Embolization** → surgical removal of RPOC	100 – 500 mL	**0**

We performed UAE prior to pregnancy termination in cases involving fetal anomalies accompanied by placental abnormalities (5 patients) or cervical pregnancy (4 patients). Minimal blood loss was observed during the subsequent gynecological procedure, and no hysterectomies were required in both cases. Additionally, 8 patients underwent embolization prior to delivery due to placental abnormalities with or without accompanied fetal anomalies. No hysterectomies were required despite slightly higher blood loss in this group, including one patient with a blood loss of 1,800 mL. Lastly, we performed embolization due to highly vascularized RPOC in 21 patients after pregnancy termination (Figure 2) and in 8 patients after delivery (Figure 3), followed by safe surgical removal, and hysterectomies were avoided in all cases within these two groups.

In the second study group (18 patients), embolization was performed as an emergency intervention to manage uncontrolled bleeding during pregnancy termination or delivery (Table 2). Among these patients, three required embolization during pregnancy termination due to excessive bleeding despite standard gynecological treatment. The embolization procedure effectively achieved hemostasis.

**Table 2.** Clinical overview of the emergency uterine artery embolization (UAE) group

**EMERGENCY UAE**	**Number of cases**	**Intervention**	**Blood loss during gynecological procedure**	**Hysterectomy**
**DURING PREGNANCY TERMINATION**				
Hemorrhage	3	Pregnancy termination → hemorrhage → **embolization**	2x 300 mL 1x 1,000 mL	**0**
**DURING DELIVERY**				
Uterine atony	10	Hemorrhage after vaginal/Cesarean delivery → intrauterine balloon tamponade → **embolization**	8x < 1,000 mL **1x > 2,000 mL** **1x > 3,000 mL**	**2**
Placental abnormalities	5	Hemorrhage after vaginal/Cesarean delivery → intrauterine balloon tamponade → **embolization**	< 800 mL	**0**

Causes of PPH included uterine atony (10 patients) and placental abnormalities (5 patients). Eight patients with uterine atony-related hemorrhage were successfully treated with UAE after standard treatment (including intrauterine balloon insertion in three patients) had failed. However, it is important to note that two cases within this group experienced ineffective hemostasis, leading to a peripartum emergency hysterectomy. The first case involved a patient with a unicornuate uterus with severe hemorrhage due to uterine atony following a cesarean section. Following unsuccessful standard treatment, emergency UAE partially alleviated the bleeding, but additional blood loss ensued, and despite extensive gynecological and endovascular efforts, an emergency hysterectomy was ultimately required as a final intervention. In the second case, the patient experienced uterine atony, rupture, and retroperitoneal hematoma following a cesarean delivery of twins, leading to significant blood loss. Despite conservative and surgical interventions followed by UAE, the bleeding persisted, and the patient progressed to hemorrhagic shock. A hysterectomy was eventually performed to save the patient's life. In the last subgroup (5 patients), we effectively managed hemorrhage related to placental abnormalities during delivery using UAE.

### Technical and clinical success

The uterine arteries were embolized bilaterally (60 patients, 94%) or unilaterally in cases involving anatomical variants (agenesis of the uterine artery) (4 patients, 6%). Nevertheless, the technical success rate was 100% as the contrast stasis was successfully achieved in all cases, as confirmed by the final angiogram.

Embolization procedures were primarily performed using absorbable gelatin sponge particles (57 patients, 89%). In a minority of cases, alternative occlusion options were employed, including a combination of gelatin sponge with microspheres (3/64), microspheres (1/64), and temporary balloon occlusion at the level of the internal iliac artery (3/64).

In the prophylactic group, a clinical success rate of 100% was achieved, indicating that all patients experienced successful outcomes. In the emergency group, the clinical success rate was 89%. Overall, hemostasis was effectively achieved in 62 out of 64 patients, resulting in a clinical success rate of 97% across the entire cohort.

In the prophylactic group, an estimated median blood loss of 200 mL was observed during the surgical intervention after embolization, and no hysterectomies were required. The performance of embolization as an emergency procedure was associated with higher blood loss. Two postpartum hysterectomies were necessary for our emergency group due to unsuccessful hemostasis despite gynecological and endovascular interventions.

### Complications

During the procedures, a single peri-procedural complication in the form of uterine artery spasm was encountered. This complication was successfully managed by administering a short-acting vasodilator (nitroglycerin), allowing for the safe continuation of the embolization without any further problems.

No major post-procedural complication was recorded in our study. However, five patients (8%) reported moderate pain in the lower abdomen in the immediate post-intervention period, which was effectively managed with oral or parenteral analgesics. Additionally, no procedure-attribut able complications were noted during the subsequent outpatient follow-up.

## Discussion

The effectiveness of UAE in managing primary and secondary postpartum hemorrhage is high, and recent literature reports 99% technical success, whereas the clinical success rates range from 87% to 95%.^1,17,20,21^ In our study, technical success in the prophylactic and emergency group was 100%. Clinical success in the prophylactic and emergency groups was 100% and 89%, respectively (overall clinical success rate of 97%). UAE was unsuccessful in achieving hemostasis in 2 patients with uterine atony following cesarean delivery, resulting in blood loss exceeding 2,000 mL and ultimately requiring a hysterectomy. In cases where embolization was unsuccessful, gelatin sponge and microspheres were used as the embolization materials. Our findings of failed endovascular therapy are consistent with those reported in the literature. Brown *et al.* and Sentilhes *et al.* described predictive factors for the failure of UAE, which include blood loss of more than 1,500 mL, DIC, large volume transfusion (> 5 red blood cell units), and cesarean delivery.^1,2^ Sugai *et al*. suggest that using a gelatin sponge as an embolic agent is associated with failure of the embolization in patients with DIC. The formation of a thrombus around the gelatin sponge depends on the patient's coagulation ability, which is impaired in DIC patients.^22^ Furthermore, Lee *et al.* observed a strong association between excessive blood loss (> 1,500 mL) accompanied by hemodynamic shock and instability and poor outcomes of UAE in a cohort of 251 patients. Nevertheless, it is still recommended that UAE should also be considered in hemodynamically unstable patients and patients with coagulopathies, but these patients require close monitoring and care.^21^

Our study suggests that UAE is a safe procedure as no major complications were recorded following the procedure. A recent systematic review of 26 studies by Zhang *et al.* reports a complication rate of 13%.^20^ The majority of complications are minor and associated with arterial puncture and angiography.^2,17^ Postembolization syndrome, characterized by transient abdominal pain, fever, nausea, and mild leukocytosis, is the most frequently reported complication, and it can be effectively managed using analgesic and anti-inflammatory medications. Other complications, such as neuropathy and organ ischemia/uterine infarction, are rare.^20^ The effects of UAE on fertility and subsequent pregnancy outcomes have not been sufficiently studied to date, and most fertility outcomes derive from UAE in the case of uterine fibroma.^4^ Existing literature reports no adverse effect on fertility in women who underwent pelvic arterial embolization in the majority of cases (91–100%).^6^ Hardeman *et al.* demonstrated no significant difference in fertility outcomes between patients who underwent UAE for severe PPH and those who did not undergo the procedure.^23^ However, some authors suggest that there may be associated with a slightly increased recurrence rate of PPH and a higher risk of first-trimester miscarriage in subsequent pregnancies.^4,5^ Although endovascular embolization preserves fertility compared to hysterectomy, further research is needed to observe the long-term effect on uterine function and future pregnancy outcomes.^6,7,8,12^

## Conclusions

Our study findings suggest that UAE is a safe and effective procedure for managing severe uterine cavity bleeding in PPH or pregnancy termination hemorrhage, as well as for reducing the risk of hemorrhage during surgical removal of highly vascularized RPOC, placental abnormalities in pregnancy termination /delivery and cervical pregnancy termination. It is a minimally invasive, uterine-sparing alternative to radical surgical treatment with hysterectomy and a promising option for patients desiring future fertility. Early cooperation between gynecologists/obstetricians and interventional radiologists may improve the clinical outcomes of UAE. The shortcoming of this study is the lack of long-term follow up to access the effect of UAE on fertility and subsequent pregnancy outcomes comprehensively.
